# X-linked Multisystem Autoinflammatory Disease With Immune Dysregulation in a Pediatric Patient: A Rare Case and Review of the Literature

**DOI:** 10.7759/cureus.94783

**Published:** 2025-10-17

**Authors:** Sara Laranja, Inês Piscalho, Matilde Caetano, Inês Coelho, Luísa Gaspar, Maria João Virtuoso

**Affiliations:** 1 Pediatrics Department, Faro’s Hospital, Faro, PRT; 2 Pediatric and Neonatal Intensive Care Department, Faro’s Hospital, Faro, PRT

**Keywords:** autoinflammatory disease, dock11 deficiency, immune dysregulation, recurrent fever, x-linked disease

## Abstract

X-linked multisystem autoinflammatory disease with immune dysregulation (ADMIDX) is a rare X-linked recessive disorder with only a few cases described in the literature. It typically presents with variable cytopenias and systemic or organ-specific autoinflammatory features. We report the case of a 10-year-old male with recurrent febrile episodes, seizures, splenomegaly, and elevated proinflammatory cytokines. Given the suspicion of an autoinflammatory condition, a genetic panel was performed, identifying a hemizygous mutation in the *DOCK11* gene, subsequently associated with ADMIDX. As the patient remains clinically stable, a conservative “watchful waiting” approach has been adopted. This report aims to raise awareness among pediatricians and emphasize the diagnostic challenges posed by this rare condition.

## Introduction

X-linked multisystem autoinflammatory disease with immune dysregulation (ADMIDX) is caused by hemizygous mutations in the *DOCK11* gene, located on chromosome Xq24 [1]. *DOCK11* is primarily expressed in hematopoietic cells, and its guanine nucleotide exchange factor (GEF) activity modulates actin cytoskeleton remodeling through activation of the small Rho GTPase CDC42 [2,3]. Germline hemizygous loss-of-function mutations in *DOCK11* result in a primary immunodeficiency due to impaired hematopoiesis and immune function [2].

ADMIDX is a rare disease, with only 11 cases reported in the literature to date [2,4], and usually manifests during infancy or early childhood. The main clinical features include variable cytopenias and systemic or organ-specific autoinflammatory symptoms such as recurrent fever, hepatosplenomegaly, arthritis, skin lesions, panniculitis, inflammatory bowel disease, or pulmonary involvement [1]. Some patients may present with immunodeficiency and recurrent infections, often accompanied by circulating autoantibodies [1]. Laboratory findings typically reflect immune dysregulation, including altered B-cell subsets and elevated proinflammatory cytokines (interleukin (IL)-2, IL-6, and tumor necrosis factor-alpha (TNF-α)) [2]. In severe cases, the disease can be fatal during childhood [1]. Therapeutic strategies remain largely experimental [5].

## Case presentation

A 10-year-old boy was referred to the Pediatric Infectious Diseases Clinic due to recurrent episodes of unexplained fever. His family history was notable for autoimmune disorders, including a father with multiple sclerosis and male siblings with neurodevelopmental conditions such as attention-deficit hyperactivity disorder and intellectual disability. No consanguinity was reported.

The patient was born prematurely at 35 weeks and three days of gestation via an emergency cesarean section due to placental abruption. He required neonatal intensive care for respiratory distress syndrome and phototherapy for neonatal jaundice. Subsequent follow-up revealed normal growth and psychomotor development. At the age of three years, he underwent tonsillectomy, adenoidectomy, and bilateral myringotomy for recurrent tonsillitis and eustachian tube dysfunction. He remained in good health until the age of nine years, when he began experiencing recurrent febrile episodes lasting three to seven days, often without evidence of infection. Three of these febrile episodes were associated with generalized tonic-clonic seizures, leading to two hospital admissions. During the first admission, at 10 years and two months of age, blood tests showed mild microcytosis without anemia, leukocytosis with neutrophil and monocyte predominance, and low C-reactive protein levels, with no additional abnormalities (Table 1). Both the electroencephalogram (EEG) and electrocardiogram results were normal. The patient was discharged after 24 hours of observation with outpatient follow-up with Pediatric Neurology.

**Table 1:** Laboratory findings during the first and second admissions to the pediatric ward. MCV = mean corpuscular volume; TWBC = total white blood cell count; ANC = absolute neutrophil count; AMC = absolute monocyte count; AST = aspartate aminotransferase; ALT = alanine transaminase; CK = creatine kinase; BUN = blood urea nitrogen; CRP = C-reactive protein

Laboratory test	First admission	Second admission	Reference range
Hemoglobin (g/L)	132	134	115–155
MCV (fL)	75.9	76.7	77.0–95.0
TWBC (/L)	14.9 × 10^9^	20.1 × 10^9^	5.0–13.0 × 10^9^
ANC (/L)	12.6 × 10^9^	16.9 × 10^9^	2.0–8.0 × 10^9^
AMC (/L)	1.2 × 10^9^	1.2 × 10^9^	0.2–0.8 × 10^9^
Platelets (/L)	365 × 10^9^	348 × 10^9^	180–400 × 10^9^
AST (UI/L)	29	Hemolysed	5–34
ALT (UI/L)	21	15	<55
CK (UI/L)	87	-	30–200
Calcium (mg/dL)	9.9	10.3	8.80–10.80
Sodium (mmol/L)	135	136	135–145
BUN (mg/dL)	14	15	7.0–16.8
Creatinine (mg/dL)	0.6	0.5	0.3–0.7
CRP (mg/L)	9	4	<5

Three months later, he was readmitted with the same presentation. Repeat blood tests revealed similar findings: mild microcytosis, neutrophilic and monocytic leukocytosis, and negative C-reactive protein (Table 1). The EEG remained normal. A lumbar puncture was performed, revealing a mild low protein level, significantly elevated IL-1β, and a slight increase in IL-6 (Table 2). A multiplex polymerase chain reaction panel for meningitis and encephalitis pathogens was negative. He was again discharged after 24 hours with continued follow-up with Pediatric Neurology.

**Table 2:** Cerebrospinal fluid findings during the second admission. IL-1β = interleukin-1-beta; IL-2 = interleukin-2; IL-6 = interleukin-6

Laboratory test	Patient’s value	Reference range
White blood cell count (cell/mm³)	4	<19
Glucose (mg/dL)	63	40–70
Total proteins (mg/dL)	13	15.0–45.0
IL-1β (pg/mL)	7.8	<1.0
IL-2 (pg/mL)	26.6	<1,000
IL-6 (ng/L)	7.0	<6.5

At his first Infectious Diseases Clinic appointment, at age 10 years and seven months, the patient was asymptomatic and had no abnormal findings on physical examination. His anthropometric parameters were within normal percentiles (Table 3).

**Table 3:** Patient’s growth percentiles at the initial Infectious Disease consultation.

Parameter	Values	Percentile
Weight	33.5 kg	P60
Height	136 cm	P25
Body mass index	18.1 kg/m²	P73

A general laboratory workup was unremarkable, including an erythrocyte sedimentation rate of 2 mm/hour (reference range <10 mm/hour). However, serum amyloid A levels were significantly elevated during febrile episodes and normalized outside of these periods (Table 4). A genetic panel for autoinflammatory diseases identified a heterozygous variant in the *UNC13D* gene, c.62_65delinsGTCTT (p.(IIe21Serfs*51)), and a hemizygous variant in the *DOCK11* gene, c.243C>T (p.(?)), described at the time as “undetermined clinical significance.” Abdominal ultrasound revealed mild splenomegaly.

**Table 4:** Serum amyloid A levels during and outside of febrile episodes.

Laboratory test	Outside febrile episodes	During febrile episodes	Reference range
Amyloid A (mg/L)	2.2	668	<10

The patient remains under multidisciplinary follow-up (Immunology and Rheumatology), currently managed with symptomatic treatment, and under ongoing consideration for initiation of immunomodulatory therapy.

## Discussion

*DOCK11* deficiency is an emerging X-linked inborn error of immunity characterized by immune dysregulation, systemic inflammation, and hematopoietic abnormalities. It is part of a group of rare disorders known as immune-related actinopathies, which result from disruptions in actin cytoskeleton remodeling, a process essential for immune cell activation, migration, and communication [2].

Functional and genetic studies in both clinical cases and animal models have demonstrated that *DOCK11* acts as a GEF for CDC42, a Rho GTPase pivotal to cytoskeletal reorganization [4]. Loss-of-function mutations in *DOCK11* impair CDC42 activation, leading to defective lymphocyte migration, abnormal T-cell activation, dysregulated cytokine production (notably IL-2, IL-6, and TNF-α), and impaired erythropoiesis [2,3].

Clinical manifestations of *DOCK11* deficiency are heterogeneous, often reflecting immune dysregulation. Reported features are summarized in Table 5 and span multiple domains, including autoimmunity, inflammation, hematologic abnormalities, and immunodeficiency.

**Table 5:** Overview of clinical manifestations reported in DOCK11 deficiency.

Clinical domain	Associated features
Autoimmunity	Autoimmune cytopenias (accelerated destruction of platelets or red blood cells induced by autoantibodies), systemic lupus erythematosus, organ-specific diseases (endocrinopathies, enteropathies, autoimmune hepatitis), and severe polyarticular juvenile arthritis [4]
Inflammation	Severe inflammatory syndrome [4,6]. Aseptic disease consistent with autoinflammation, including intermittent episodes of self-limited malaise and fever, persistently elevated acute-phase reactants, and recurrent severe pneumonia in the absence of detection of relevant infectious agents[5]. Clinical indicators of autoinflammation may be present[5]
Hematologic	Normocytic anemia (rarely microcytic)[2], thrombocytopenia or thrombocytosis[4], and functional thrombopathy (normal platelet count and size, but with abnormal morphological modifications)[6]
Immunologic	Recurrent infections, hypogammaglobulinemia[5], elevated inflammatory cytokines, lower percentage of memory B cells[4], and lower percentages of CD56 bright natural killer (NK) cells and late NK cells[4]
Other	Muscle hypotonia, developmental delay, bronchiectasis, renal amyloid A amyloidosis, and hepatosplenomegaly [2,5]

Therapeutic strategies remain largely experimental. Corticosteroids and IL-1 inhibitors such as anakinra have shown potential in mitigating inflammation. Intravenous immunoglobulin may be beneficial in patients with concomitant immunodeficiency. While gene therapy and hematopoietic stem cell transplantation are theoretical treatment options for severe phenotypes, they remain investigational and untested in this context [5].

Our patient’s clinical picture, recurrent fever, mild splenomegaly, systemic inflammation, and elevated proinflammatory markers (IL-1β, IL-6, serum amyloid A), aligns with the phenotype of patients exhibiting complete loss of *DOCK11* expression [2,3,5]. The association of amyloid A elevation with *DOCK11*-related inflammation has been described in prior reports and reflects the chronic inflammatory burden of the disease [2].

Although recurrent infections are a recognized feature in some *DOCK11*-deficient individuals, particularly those with nonsense mutations abolishing protein expression [2,3], other cohorts (notably those with residual protein function due to missense mutations) do not consistently report infectious susceptibility [4,6]. This observation underscores a possible genotype-phenotype correlation, wherein complete loss-of-function variants are more closely associated with autoinflammation and infections, while missense variants are more often linked to autoimmunity [5].

Seizures, while not classically reported in *DOCK11* deficiency, were observed in our case. Although neurological symptoms such as hypotonia and developmental delay have been described [2], seizures may represent a rarer manifestation or a secondary effect of systemic inflammation.

The genetic panel for autoinflammatory diseases in this patient was conducted in November 2023. The first description of *DOCK11*/*ADMIDX* in the OMIM database was published in June 2023, with a subsequent update in November 2023 [1]. This temporal overlap may account for the variant being reported as of “undetermined clinical significance.”

Of additional complexity is the finding of a heterozygous *UNC13D* variant. Homozygous or compound heterozygous mutations in this gene are known to cause familial hemophagocytic lymphohistiocytosis type 3, a hyperinflammatory syndrome characterized by fever, cytopenias, hepatosplenomegaly, and sometimes seizures [7]. Laboratory findings may include hyperferritinemia, hypertriglyceridemia, and reduced fibrinogen [7], although these were absent in our patient. As the variant was heterozygous, it is not considered disease-causing but could have a modifying effect.

Importantly, in the cohort described by Block et al., no cases of hemophagocytic lymphohistiocytosis were reported among patients with *DOCK11* deficiency, further suggesting phenotypic distinctions between isolated *DOCK11* mutations and hemophagocytic lymphohistiocytosis syndromes related to cytotoxic pathway defects [6].

The concurrent presence of variants in both *DOCK11* and *UNC13D* raises the possibility of synergistic or modifying genetic interactions that may influence the clinical presentation. Future studies are needed to explore such potential digenic inheritance or gene-gene interactions in autoinflammatory diseases.

## Conclusions

*DOCK11* deficiency exemplifies the diagnostic challenges associated with novel monogenic autoinflammatory syndromes. This case reinforces the importance of including *DOCK11* in next-generation sequencing panels when evaluating pediatric patients with unexplained systemic inflammation, early-onset autoimmunity, or atypical cytopenias. Our report highlights the need for increased awareness of *DOCK11* deficiency among clinicians and researchers, especially considering its clinical overlap with other inborn errors of immunity, such as hemophagocytic lymphohistiocytosis. As more cases are identified and molecular characterization advances, collaboration across registries and international research networks will be crucial to delineate the phenotypic spectrum, clarify genotype-phenotype correlations, and guide therapeutic decision-making. Furthermore, the co-occurrence of pathogenic variants in both *DOCK11* and *UNC13D* in this patient underscores the potential complexity of genetic contributions to autoinflammatory syndromes and the value of comprehensive genetic evaluation in diagnostically challenging cases.
